# Prevalence of African swine fever virus in apparently healthy domestic pigs in Uganda

**DOI:** 10.1186/1746-6148-9-263

**Published:** 2013-12-26

**Authors:** David Kalenzi Atuhaire, Mathias Afayoa, Sylvester Ochwo, Savannah Mwesigwa, Frank Norbert Mwiine, Julius Boniface Okuni, William Olaho-Mukani, Lonzy Ojok

**Affiliations:** 1 College of Veterinary Medicine, Animal resources and Biosecurity, Makerere University, P.O.BOX 7062, Kampala, Uganda; 2 African Union-Interafrican Bureau for Animal Resources, P.O.BOX 30786, Nairobi, Kenya; 3 National Agricultural Research Organization, National Livestock Resources Research Institute, P.O.BOX 96, Tororo, Uganda

**Keywords:** African swine fever virus, ELISA, PCR, Prevalence, Seropositive, Slaughter pigs, Surveillance

## Abstract

**Background:**

African swine fever (ASF) is a contagious viral disease which can cause up to 100% mortality among domestic pigs leading to serious socio-economic impact on people’s livelihoods. ASF is endemic in Uganda and there is paucity of information on the epidemiology of the disease. The major aim of this study was to determine the seroprevalence and prevalence of African swine fever virus (ASFV) in apparently healthy slaughter pigs at Wambizi slaughterhouse in Kampala city, Uganda. We also estimated the presence of ASFV antibodies and circulating viral antigens in pigs from selected districts of Uganda during targeted surveillance. We analysed 540 and 181 blood samples collected from slaughter pigs and pigs from targeted surveillance districts respectively.

**Results:**

The prevalence of ASFV in slaughter pigs was 52.96% (95% CI, 48.75-57.14) and 11.5% (95% CI, 9.06-14.45) by ELISA and PCR respectively. In surveillance districts, the proportion of ASFV positive pigs was 53.59% (95% CI, 46.33-60.71) and 0.55% (95% CI, 0.1-3.06) by ELISA and PCR respectively.

**Conclusion:**

The study has found out a high seroprevalence of ASFV antibodies in apparently healthy slaughter pigs and also a high proportion of ASFV antibody seropositive pigs in surveyed districts in Uganda indicating exposure to ASFV. However, there was a lower prevalence of ASFV infection implying that there could be low virulent strains of ASFV circulating in domestic pigs in Uganda which requires further investigation.

## Background

African swine fever (ASF) is a viral disease with a devastating impact on the pig industry in sub-Saharan Africa [1]. The disease is caused by double-stranded DNA virus with an icosahedral symmetry that belongs to genus *Asfivirus* and family *Asfarviridae*[2]. ASF virus replicates primarily in cells of the reticulo-endothelial system [3], and consequently blood, lymph node, spleen, liver and tonsil are the preferred specimens for laboratory examination [4]. The epidemiology of ASF is complex, transmission is direct and vector-borne, and the disease has well recognized sylvatic and domestic cycles [5]. ASF is highly contagious and is transmitted by direct contact between infected pigs and susceptible ones or by contact with infectious secretions/excretions [4]. The virus is highly resistant in tissues and the environment, contributing to its transmission over long distances through swill feeding and fomites (e.g., contaminated material, vehicles or visitors to pig premises) [4].

Uganda has the largest pig population in Eastern Africa standing at 3.2 million [6]. Pig farming is one of the fastest growing livestock activities in the rural areas of Uganda and has become very attractive throughout the country as a means of increasing food, income and employment [7,8]. But ASF is an economically important and frequently lethal disease of domestic pigs which has hampered the development of the pig industry in Uganda [8] and other affected countries [1].

In endemic areas, diagnosis may be based on detection of antibody but in new introductions of disease it is preferable to detect the virus. Procedures, which have been used for ASF virus antigen detection, include the traditional haemadsorption test (HAD; [9], radioimmunoassay (RIA; [10];, direct immunofluorescence [11] and polymerase chain reaction (PCR; [12-14] and recently LAMP [15]. Immunocytochemistry and *in situ* hybridization have been described in studies of disease pathogenesis [16], but these techniques are not ideally suited to routine diagnosis [17]. Serological examinations may be the best way to detect pigs infected with ASF virus [18]. Recently, an ELISA was developed for the serodiagnosis of ASFV in Africa independent of the geographical origin of the sera based on the p30 recombinant protein (p30r) obtained from an East African viral isolate (Morara Strain) [19]. However, the p30r was not subjected to samples from Uganda and Kenya where genotype IX is known to circulate [19].

Following ASF outbreaks, antibodies can persist in recovered pigs for long periods after infection, sometimes for life [20]. Previous experimental studies on persistence of ASFV revealed that viral DNA is detectable in peripheral blood mononuclear leukocytes at greater than 500 days post infection by PCR assay, although it was not possible to isolate the infectious virus from that sample [21]. This indicates that monocytes/macrophages may be persistently infected with ASFV [22]. Although no long-term carrier state has been demonstrated, these pigs were shown to remain infected for up to several weeks [23], and can transmit the disease to other susceptible pigs. Sub-clinically infected, chronically infected or recovered pigs are likely to play an important role in the epidemiology of the disease, for disease persistence in endemic areas as well as for causing sporadic outbreaks or introduction into disease-free zones [4,24-26]. In endemic areas, mortality rates have decreased and sub-clinical or chronic ASFV infections have become more frequent [24,27,28]. Pigs infected with isolates of low virulence may seroconvert without symptoms, abort or develop chronic African swine fever [20,29].

The major aim of this study was to determine the seroprevalence and prevalence of ASFV in apparently healthy pigs slaughtered in Wambizi slaughter house in Kampala city. The study also aimed at estimating the presence of ASFV antibodies in pigs from selected districts with no active ASF outbreak in order to give an insight in presence of the antibodies and circulation of the viral antigens in apparently health domestic pigs.

## Methods

### Study sites

**Figure 1:**
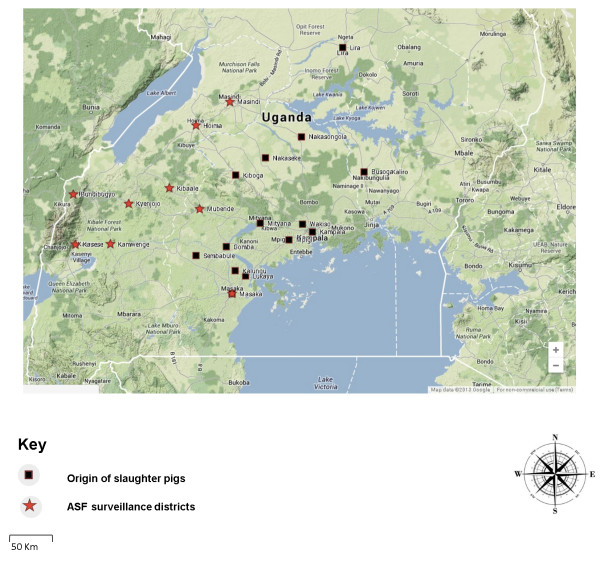
Map of Uganda showing origins of slaughter pigs and districts of targeted African swine fever surveillance.

The study was carried out in Wambizi slaughterhouse, the largest pig slaughterhouse in Kampala City, run by a farmer cooperative located in Nalukolongo. This slaughter house was chosen because it is the largest in Kampala and receives pigs from most regions in the country. In addition, targeted surveillance was done in 10 selected districts of Uganda. These districts were conveniently selected for purposes of this study. The districts included Masaka, Mityana, Mubende, Kyenjojo, Kamwengye, Kasese, Bundibugyo, Kibaale, Hoima and Masindi (Figure 1). Sample villages and pig herds were identified with the advice of the respective District and sub county Veterinary officers, and farmers’ consent was obtained before pig sampling.

### Study design

A cross-sectional study to estimate ASFV prevalence in slaughter pigs was conducted for a period of one year. For stratified random sampling at the slaughterhouse, we used the formula k = N/n [30], where k is the k^th^ element, *n* is the sample size, and *N* is the population size. We were collecting 20–25 blood samples (n) per visit to the slaughterhouse. Since it was estimated that an average of 100 pigs (N) were slaughtered per day it therefore meant that the k value was approximately 5. We therefore sampled every fifth slaughtered pig and recorded the origin (region and district). The information on the origins was provided by the traders at the slaughter house though some pigs sampled were from an unknown origin. During every visit to the abattoir each pig to be slaughtered had an equal chance/probability of being selected. Only apparently healthy pigs were sampled. A convenient sampling strategy was used during surveillance to select pig herds from which blood samples were collected.

### Sample size determination

To estimate the prevalence of ASFV in apparently healthy slaughter pigs at Wambizi slaughter house, the sample size for pigs that achieved 5% level of precision at 95% confidence level was calculated using the formula described by [31]. n = Z^2^ X P (1-P)/d^2^; Where; n = Sample size, Z = 1.96 at 95% confidence level, P = Probable prevalence of ASFV in pigs slaughtered at Wambizi (taken as 6%), d = Standard error (taken as 2%). However, 540 blood samples were successfully analysed in this study. In addition, we collected 181 blood samples from the 10 districts where targeted surveillance was conducted.

### Sample collection

Two visits to the slaughter house were made per month and blood samples collected. Blood was collected from the jugular vein into vacutainers for serum and EDTA tubes. Serum was then separated in the laboratory and stored at -20°C till used in the ELISA assay. An aliquot of the blood sample was stored at -20 °C till used for DNA extraction for PCR.

### ELISA assay

An indirect ELISA was performed to determine the presence of ASF virus antibodies in pig sera collected from Wambizi slaughterhouse and selected districts in Uganda. The procedure was as described previously [32,33]. We used a VP73 antigen (a purified protein extract from ASFV received as a donation from the OIE reference laboratory, Ingenasa, Spain), and a secondary antibody (Goat anti-porcine IgG labeled with Horse-raddish peroxidase) diluted 1:5000 in dilution buffer. Optical Densities (ODs) were measured at 490 nm. The cut offs were determined as previously described [32,33]. The cut-off was then used to determine whether a sample was positive, negative or inconclusive [32,33].

### DNA extraction

Viral DNA was extracted directly from 200 μl aliquots of blood collected in EDTA tubes using the DNeasy Blood and tissue kit (QIAGEN®, USA).

### Genomic amplification

A 278 bp region corresponding to the central portion of the p72 gene was amplified using the diagnostic primers, primer 1 (5′-ATGGATACCGAGGGAATAGC-3′) and primer 2 (5′-CTTACCGATGAAAATGATAC-3′) to confirm the presence of ASFV DNA [34].

**Figure 2:**
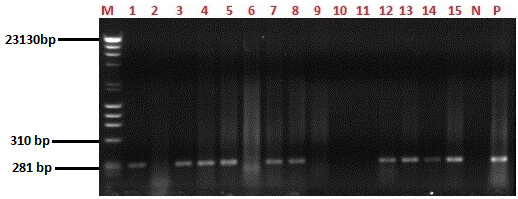
**PCR amplification of the p72 gene.** A 1.5% Agarose gel showing the representative results obtained with the diagnostic primers targeting the central portion of the p72 gene of African swine fever virus. Lane M; λ DNA-Hind III and ϕx174 DNA-Hae III mix (Finnzymes) Molecular weight DNA marker, Lanes 1,3,4,5,7,8,12,13,14 and15 are selected positive samples with approximately 278 bp band size. Lanes 2,6,9,10 and 11 are negative samples. Lane N is negative control and Lane P is a positive control.

Genomic amplification was performed in a 50 μl volume in the presence of 200 μmol/L dNTP, 0.25 μmol/L of each primer, 2.5U of thermostable *Taq* DNA polymerase and 3 μl of DNA extract. The template was amplified following 40 cycles of denaturation at 96 °C for 12 s, annealing at 53°C for 20 s and extension at 70°C for 30s. Amplification products were loaded on a 1.5% agarose gel and run against a 100 bp DNA ladder. Once sufficient electrophoretic separation was obtained, the products were visualized by UV irradiation and stained with ethidium bromide. The gels were photographed in order to provide a soft copy result for the diagnostic products and a representative gel is shown in Figure 2.

## Results

### Proportion of ASFV seropositive slaughter pigs by Indirect ELISA

The proportion of ASFV seropositive slaughter pigs and their origins is summarized in Table 1. Most of the pigs slaughtered (42.6%) at the slaughterhouse originated from Masaka district. With the exception of Busoga, Lango and Unknown, all the other districts from where the slaughter pigs originated fall in central Uganda. This implies that 81.9% of all the slaughtered pigs originated from central Uganda. The overall proportion of slaughter pigs that were seropositive for ASFV over a one year period was 52.96% (95% CI, 48.75-57.14).

**Table 1:** Proportion of ASFV seropositive slaughter pigs at Wambizi slaughterhouse (February 2012- January 2013)

**Origin of pigs**	**Total sampled**	**Number positive**	**Number inconclusive**	**Number negative**	**Proportion of seropositive pigs**
Busoga	54	35	4	15	64.7
Gomba	8	3	2	3	33.33
Kalungu	47	15	3	29	32
Kampala	38	5	2	31	11.76
Kiboga	18	8	1	9	42.86
Lango	23	11	1	11	47.37
Lukaya	19	8	1	10	42.86
Masaka	230	130	21	79	56.5
Mityana	27	27	0	0	100
Mpigi	17	2	1	14	9.09
Nakaseke	5	5	0	0	100
Nakasongola	9	9	0	0	100
Sembabule	10	5	0	5	50
Unknown	21	17	1	3	80.95
Wakiso	14	6	2	6	42.86
**Total**	**540**	**286**	**39**	**215**	**52.96**

### Proportion of pigs with ASFV antibodies during field surveillance by indirect ELISA

The proportion of pigs positive for ASFV antibodies during field surveillance is summarized in Table 2. The overall proportion of ASFV seropositive pigs during surveillance was 53.59% (95% CI, 46.33-60.71).

**Table 2:** Proportion of ASFV seropositive pigs during surveillance in selected districts of Western and Central Uganda

**District sampled**	**Total sampled**	**Number positive**	**Number inconclusive**	**Number negative**	**Proportion of seropositive pigs (%)**
Bundibujo	12	11	1	0	91.67
Hoima	6	4	1	1	66.67
Kamwenge	9	7	2	0	77.78
Kasese	8	8	0	0	100
Kibaale	16	11	1	4	68.75
Kyenjojo	2	1	1	0	50
Masaka	95	33	25	37	34.74
Masindi	13	8	2	3	61.54
Mityana	7	6	1	0	85.71
Mubende	13	8	3	2	61.54
**Total**	**181**	**97**	**37**	**47**	**53.59**

### Prevalence of ASFV by diagnostic PCR

A total of 62 blood samples out of 540 analyzed tested positive for ASFV using the OIE recommended diagnostic PCR (Table 3). ASFV prevalence was 11.5% (95% CI, 9.06-14.45) among the slaughter pigs sampled over a one year period (February 2012 to January 2013). Out of the 62 PCR positive samples 43 were positive for ASFV antibodies, 12 were negative while 7 were inconclusive by ELISA. Only one sample [0.55% (95% CI, 0.1-3.06)] was positive by PCR out of the 181 pigs sampled during field surveillance.

**Table 3:** Proportion of PCR positive pigs and their areas of origin in Uganda

**Origin of pigs**	**Number of pigs sampled**	**Number of PCR positives**	**Proportion of PCR positive pigs (%)**
Busoga	54	11	20.4
Kalungu	47	7	14.9
Kampala	38	4	10.5
Lango	23	7	30.4
Masaka	230	27	11.7
Nakaseke	5	1	20
Nakasongola	9	2	22.2
Sembabule	10	2	20
Unknown	21	1	4.8
**Total**	**540**	**62**	**11.5**

### Monthly ASFV detection in slaughter pigs

ASF was detected most (42.2%) in the month of March followed by November (20%) and, April (15.6%), May (13.3%), June, December and January (11.1%), August (6.5%), September (4.4%) and July (2.2%). There was no ASFV detected in slaughter pigs in the months of February and October. The distribution of the sample size from the different areas from which at least one sample tested positive by PCR is shown in Table 4.

**Table 4:** Number of ASF PCR positive domestic pigs slaughtered in Wambizi slaughterhouse by Month and origin

**Origin of pigs**	**Months (Feb 2012-Jan 2013)**												
	F	M	A	M	J	J	A	S	O	N	D	J	
Busoga	_	_	2	4	_	_	_	_	_	3	_	2	(11)
Kalungu	_	_	_	_	4	_	_	_	_	2	_	1	(7)
Kampala	_	_	_	_	_	1	2	_	_	_	1	_	(4)
Lango	_	2	4	_	_	_	_	_	_	_	1	_	(7)
Masaka	_	17	_	2	_	_	1	_	_	4	2	1	(27)
Nakaseke	_	_	_	_	_	_	_	1	_	_	_	_	(1)
Nakasongola	_	-	1	-	_	_	_	_	_	_	_	1	(2)
Sembabule	_	_	_	_	1	_	_	_	_	_	1	_	(2)
Unknown	_	_	_	_	_	_	_	1	_	_	_	_	(1)
**Total**	**0**	**19**	**7**	**6**	**5**	**1**	**3**	**2**	**0**	**9**	**5**	**5**	**(62)**

## Discussion

The aims of the current study were to determine the prevalence of ASFV in slaughter pigs in Wambizi slaughterhouse, Kampala city and to determine the proportion of ASFV seropositive domestic pigs during surveillance in areas with no active ASF outbreak in order to give an insight in presence of the antibodies and circulation of the viral antigens in apparently health domestic pigs in Uganda. The disease is endemic in the country [35] and outbreaks have been reported since 2001 [8]. The inability of ASFV to induce neutralizing antibodies has hampered the prevention and control of the disease by vaccination and to date there is no vaccine for ASF. In the absence of effective vaccines, control is based on rapid laboratory diagnosis and the enforcement of strict sanitary measures [36]. In ideal situations diagnosis of ASF should be carried out through a combination of tests including the detection of viral genome by PCR, the detection of viral antigen by antigen ELISA or FAT and the detection of virus through virus isolation. This is not however possible in many countries like Uganda in which ASF is endemic, partly due to high cost of molecular diagnostic tools and the difficulty and cost of carrying out virus isolation. Cheaper assays are available for the detection of ASF antigen, such as the antigen ELISA and FAT; however, these assays have a reduced sensitivity compared to PCR. In this study the OIE recommended diagnostic PCR and ELISA were used to determine the prevalence of ASFV. The early appearance and long-term persistence of antibodies and the lower cost compared to PCR-based methods emphasize the relevance of antibody detection techniques in the control of the disease in affected countries [37].

The majority (81.9%) of the pigs slaughtered at Wambizi slaughterhouse originated from the central region of Uganda. This agrees with a previous study carried out in the same slaughterhouse where 68% of the slaughtered pigs originated from the central region [38]. This could be because Wambizi slaughterhouse is located in the central region or even because this region has the highest number of pigs in Uganda [6].

In this study the overall seroprevalence of ASFV in slaughter pigs over a one year period was found to be 52.96% (95% CI, 48.75-57.14). This is a higher seroprevalence compared to previous studies in Uganda [39-42] and other countries [43,44]. A similar study on slaughter pigs in Mubende district in central Uganda found a much lower prevalence of 0.2% [39]. Another study carried out in Rakai district in Southern Uganda reported a seroprevalence of 2.1% [40]. However, a previous study on 39 porcine serum samples obtained from central Ugandan districts revealed that none of them were antibody positive for ASF using the OIE recommended ELISA [42]. In a study carried out in Gulu district in Northern Uganda on 6 pigs one year post ASF outbreak survival only 3 pigs were found to have antibodies against ASFV [41] suggesting persistence of antibodies and/or circulation of a low virulent ASFV. The results obtained in this study are strikingly different from previous similar studies in Uganda. This could be attributed to the fact that pigs from which blood samples were collected in this study were from diverse geographical origins (Figure 1) in comparison to previous studies in Uganda that sampled only in one district [39-41]. Contrary, the current study was carried out for a period of 12 months in slaughter domestic pigs while other previous studies in Uganda were of a shorter period of time [40-42]. Furthermore, these previous studies [40-42] used a very low sample size compared to this study. Nevertheless, a study by Muwonge and others (2012) used a higher sample size but the pigs were from one geographical locality of Mubende district in Central Uganda [39].

In addition, the proportion of ASFV antibody positive domestic pigs during field surveillance was 53.59% (95% CI, 46.33-60.71). This is close to the observed seroprevalence (52.96%) of slaughter pigs in this study. Since outbreaks occur sporadically in most parts of Uganda [8], it could also be that following an acute phase of ASF, the pigs that survive have detectable levels of antibodies in serum against the ASFV after 7–12 days post development of the first clinical symptoms [20]. These antibodies persist for long time, probably for life [20]. The higher seroprevalence obtained in this study shows that approximately half of the pigs slaughtered have been exposed to ASFV.

The overall ASFV prevalence determined by PCR among the slaughtered pigs sampled over a one year period was found to be 11.5% (95% CI, 9.06-14.45). The proportion of apparently healthy pigs that were ASFV antigen positive during field surveillance was 0.55%. This could be that this pig had an active infection or was a survivor of an acute infection. The low proportion of ASFV antigen positive pigs could also be attributed to the effect of temperature differences from field collection sites during transportation to the laboratory which could probably have degraded the viral DNA. The use of FTA cards would have mitigated this problem. A related study in Rakai district in Southern Uganda found a prevalence of 3.3% in apparently healthy domestic pigs [40]. In this study ASFV was detected in slaughter pigs in all the months except in February and October with the majority detected in the month of March. A previous study on the epidemiology of ASF outbreaks in Uganda has reported ASF in all months between 2001 and 2012 with the majority of the outbreaks occurring in the dry season [8]. Uganda experiences two dry seasons of December to February and June to August [45]. The findings in this study agree with the previous study that showed a sporadic pattern of ASF occurrence, the difference being the increase of ASF outbreaks at the peaks of the dry seasons that was not observed in the current study.

Out of the 62 PCR positive samples 43 were positive for ASFV antibodies. These pigs could have been chronic carriers, subclinically infected and/or recently infected with the virus but not showing the clinical signs. The presence of ASFV antibodies and ASFV DNA in apparently healthy domestic pigs suggests that circulation of ASF virus in the pig population in Uganda is a critical issue and that these pigs may play a great role in the maintenance of ASF in the country and may be spread by in-apparent carriers to other virus free swine. The detection of ASF specific antibodies is indicative of previous infection [37], and since antibodies are produced from the first week of infection and persist for long periods they are appropriate markers for the diagnosis of the disease [33].

Future studies should aim at determination of IgM titres which would be useful for epidemiological studies in endemic areas since positive detection of antibodies would indicate previous exposure and not necessarily a current infection. In such cases, the IgM/IgG ratio would distinguish between exposed and recently infected pigs [46]. Other recently developed ASFV recombinant proteins for serodiagnosis of ASF need to be validated for use in endemic countries like Uganda.

In this study we had limitations that could have introduced bias. The convenient sampling approach used during surveillance was a drawback in that the samples collected were not representative of the total pig populations in the selected districts and this could not be used to estimate the ASFV prevalence. Furthermore, we experienced logistical constraints; the reason why sample collection at the slaughter house was for only two days in a month. The study could have probably benefited more if samples were collected daily for the entire year.

## Conclusion

In conclusion, the study has found out a high seroprevalence of ASFV in apparently healthy slaughter pigs and also a high proportion of ASFV seropositive pigs in surveyed districts in Uganda. The fact that most seropositive pigs were PCR negative indicates that they were exposed to ASFV but had recovered from the infection, which is suggestive of circulation of low virulent strains which requires further investigation.

## Competing interests

The authors of this paper do not have any financial or personal relationship with other people or organizations that could inappropriately influence or bias the content of the paper. The authors declare that they have no competing interests.

## Authors’ contributions

DKA contributed to the conception of the idea, design, data collection, laboratory studies, drafting and writing of the manuscript. MA contributed on data collection, laboratory studies and manuscript preparation. SO contributed to laboratory studies, data analysis and drafting of the manuscript. SM contributed to data collection, data analysis and writing of the manuscript. FNM contributed to data collection, laboratory studies and writing of the manuscript. JBO contributed to conception of the idea, data collection and writing of the manuscript. WO contributed to conception of the idea, design and writing of the manuscript. LO contributed to conception of the idea, design, data collection and writing of the manuscript. All read and approved the manuscript.

## Authors’ information

DKA and MA are PhD candidates working on African swine fever in Uganda. SO is an MSc student working on African swine fever diagnostics. SM is a PhD student working on Trypanosomiasis. FNM is a PhD holder with expertise in animal viruses. JBO is a PhD holder with expertise in Veterinary diagnostics. WO is a PhD holder and has researched widely in the livestock sector. LO is a PhD holder and a Professor of Veterinary Pathology with vast experience on diagnosis of livestock diseases.
